# ChatGPT in reducing vaccine hesitancy and enhancing vaccine acceptance: correspondence

**DOI:** 10.1590/1806-9282.20240734

**Published:** 2024-08-16

**Authors:** Hinpetch Daungsupawong, Viroj Wiwanitkit

**Affiliations:** 1 Private Academic Consultant – Phonhong, Lao People's Democratic Republic.; 2 Saveetha University, Saveetha Institute of Medical and Technical Sciences, Saveetha Dental College and Hospitals, Department of Research Analytics – Chennai, India.

Dear Editor,

"Application of ChatGPT in reducing vaccine hesitancy and enhancing vaccine acceptance: Hope or myth?^1^" is an interesting article. In conclusion, vaccine hesitancy has been effectively addressed by using ChatGPT, a text-generative artificial intelligence tool, which clears up myths and provides correct facts. This has been especially important during the COVID-19 pandemic when efforts to promote public health have been severely hampered by reluctance and false information. ChatGPT has the capacity to inform and empower people to make knowledgeable vaccination decisions, which could ultimately result in a rise in vaccine adoption and better public health outcomes.

But even with all of its apparent advantages, ChatGPT is not without flaws. Depending on the version utilized, it can give false information, which could cause confusion and even injury if people depend only on its answers when making medical decisions. Furthermore, ChatGPT might not have the most recent information after a certain point, which would restrict its usefulness in addressing pressing problems and changing healthcare difficulties. Moreover, the instrument might not encompass the subtleties of personalized patient care and might not provide the comprehensiveness and precision necessary for intricate medical situations.

Going forward, it is imperative to recognize and rectify these shortcomings in ChatGPT in order to optimize its efficacy in mitigating vaccine reluctance. Future developments can include adding real-time updates and making sure that the data the tool provides are accurate and dependable. The ability of the tool to handle a variety of patient groups and customize responses to suit each person's requirements and preferences should also be improved. Partnerships between medical practitioners, IT specialists, and public health authorities can maximize ChatGPT's usage by encouraging vaccine acceptance and successfully dispelling false information.

In conclusion, even though ChatGPT has demonstrated promise in reducing vaccination hesitancy and encouraging vaccine acceptance, it is critical to acknowledge its limits and keep enhancing and perfecting the tool in order to meet the changing demands of the medical field. By utilizing ChatGPT and other AI-powered healthcare tools, we can improve communication, give people accurate information, and encourage them to make well-informed immunization decisions. ChatGPT has the potential to significantly influence how healthcare is delivered in the future and how public health activities are carried out with additional development and cooperation.
